# Description of four new species of armoured spiders (Araneae, Tetrablemmidae) from Sumatra, Indonesia

**DOI:** 10.3897/zookeys.820.29363

**Published:** 2019-01-29

**Authors:** Riko Fardiansah, Nadine Dupérré, Rahayu Widyastuti, Anton Potapov, Stefan Scheu, Danilo Harms

**Affiliations:** 1 University of Göttingen, J.F. Blumenbach Institute of Zoology and Anthropology, Untere Karspüle 2, 37073 Göttingen, Germany; 2 Zoological Museum, Center of Natural History, Universität Hamburg, Martin-Luther-King-Platz 3, 20146 Hamburg, Germany; 3 Institut Pertanian Bogor – IPB, Department of Soil Sciences and Land Resources, Dramaga Campus, Bogor 16680, Indonesia; 4 University of Göttingen, Centre of Biodiversity and Sustainable Land Use, Von-Siebold-Str. 8, 37075 Göttingen, Germany

**Keywords:** *
Ablemma
*, tetrablemmids, rainforest, Southeast Asia, taxonomy, new genus record

## Abstract

Four new species of armoured spiders from Sumatra, Indonesia are described. Three species are described in the genus *Ablemma* Roewer, 1963 and one species in the genus *Brignoliella* Shear, 1978; *Ablemmaandriana***sp. n.** (male), *Ablemmacontrita***sp. n.** (male and female), *Ablemmakelinci***sp. n.** (male) and *Brignoliellapatmae***sp. n.** (male and female). The female of *Ablemmasingalang* Lehtinen, 1981 is described here for the first time. The first record of *Brignoliella* for Sumatra is also presented.

## Introduction

Tetrablemmidae are small (0.8–2 mm), cryptozoic spiders predominantly living in forest leaf litter (Jocqué and Dippenaar-Shoeman 2007); they are very diverse in Southeast Asia. Of the 131 known species in this family, 50 have been described from this region (World Spider Catalog 2018). Thirty-five species are found in Eastern and Southern Asia with the remaining species distributed around the world except in Central and Western Asia, Europe, Central America, in the Arctic and Antarctic region. The south Asian fauna of the families Tetrablemmidae and Pacullidae was first studied by Shear (1978) followed by Deeleman-Reinhold (1980) and finally in 1981, Lehtinen published a world revision that united the two families into the family Tetrablemmidae. More recently, taxonomic work has been done on the Asian fauna by Lin et al. (2012, 2017, 2018).

The family was recently redefined to include only 27 genera that belong to the sub-family Tetrablemminae, and the sub-family Pacullinae was elevated to family level including four genera (Wheeler et al. 2017).

In Sumatra, Indonesia, the family Tetrablemmidae remains poorly documented; a total of six species in four genera have been recorded on this island (Stenchly 2011, World Spider Catalog 2018): *Ablemmabaso* Roewer, 1963, *A.erna* Lehtinen, 1981, *A.singalang* Lehtinen, 1981; *Pahangalilisari* Lehtinen, 1981; *Singalangiasternalis* Lehtinen, 1981; and *Tetrablemmamardionoi* Lehtinen, 1981.

In this paper, we describe four new species of Tetrablemmidae and present the first record of the genus *Brignoliella* from Harapan and Bukit Duabelas, Jambi Province, Sumatra. The specimens were sampled from leaf litter and topsoil in lowland rainforest of Sumatra and were collected as part of the Ecological and Socio-economic Functions of Tropical Lowland Rainforest Transformation Systems project (EFForTS), which investigates consequences of converting lowland rainforest into plantation systems (Drescher et al. 2016). Extensive field surveys are being carried out in rainforest, jungle rubber agroforests, rubber plantations and oil palm plantations and the spider fauna is currently being studied in each habitat for its taxonomic and ecological composition. This is the second paper describing new species of spiders from this project, following the recent description of new species of goblin spiders (Fardiansah et al. 2018).

## Material and methods

All specimens were collected in the framework of the EFForTS project (Drescher et al. 2016). The material was collected on the plots of the Collaborative German-Indonesian Research Center (CRC990) with the following ecosystem types: primary degraded lowland rainforest, jungle rubber originating from planting rubber trees (*Heveabrasiliensis* Müll.Arg.) into lowland rainforest but predominantly composed of rubber trees, rubber monoculture, and oil palm (*Elaeisguineensis* Jacq.) monoculture. Spiders were collected with two methods: (1) by sieving litter layer from the area of 1 × 1 m and further hand collection and (2) by taking 16 ×16 cm samples of litter and the underlying top 5 cm of soil and further heat extraction; all collected specimens were placed into 70% ethanol (Barnes et al. 2014, Klarner et al. 2017). The material examined is deposited in the following institutions: Indonesian Institute of Sciences Cibinong, Indonesia (**LIPI**); Zoological Museum Hamburg, Hamburg, Germany (**ZMH**).

Specimens were examined in 75% ethanol under a Leica M125 dissection microscope. Specimens were photographed with a custom-made BK Plus Lab System by Dun, Inc. with integrated Canon camera, macro lenses (65 mm and 100 mm) and the Zerene stacking software. Female genitalia were excised using a sharp entomological needle placed on a slide in lactic acid and observed under a Leica microscope DM2500 LED compound microscope. A Leica DMC 4500 digital camera attached to the microscope was used to photograph all the structures to be illustrated. The digital photos were used to trace proportions and the illustrations were detailed and shaded by referring back to the structure under the microscope. All morphological measurements are given in millimetres. Matching of males and females can be challenging and when in doubt females were not matched. Otherwise males and females were matched based on the following criteria: (1) collected in the same sample, (2) body size and colour, and (3) abundance. Morphological nomenclature follows Lin et al. (2017, 2018).

Abbreviation used in figures are as follows:

**ALE** anterior lateral eyes;

**alg** anterolateral groove of preanal scutum;

**AME** anterior median eyes;

**as** anal scutum;

**b** palpal bulb;

**bp** embolic basal projection;

**ca** cheliceral apophysis;

**cb** cheliceral basal boss;

**cl** cheliceral lamina;

**ct** carapace tubercle;

**d** duct;

**e** embolus;

**ea** embolic apophysis;

**ip** inner genitalic plate;

**PLE** posterior lateral eyes;

**PME** posterior median eyes;

**ps** postgenital scutum;

**prs** preanal scutum;

**pls** pulmonary scutum;

**sd** sperm duct;

**sr** seminal receptacle;

**plc** posterolateral corner of preanal scutum;

**pmc** posteromedial corner of preanal scutum.

## Taxonomy

### Family Tetrablemmidae O. Pickard-Cambridge, 1873

#### Genus Ablemma

Taxon classificationAnimaliaAraneaeTetrablemmidae

Roewer, 1963

##### Type species.

*Ablemmabaso* Roewer, 1963

##### Diagnosis.

The genus *Ablemma* most resembles the genera *Singaporemma* Shear, 1978 and *Sulaimania* Lehtinen, 1981 but it can be distinguished from both genera by the combination of the following characters: 6, 4 or 2 eyes; sternum reticulate (Fig. 2); male carapace without clypeal horn (Figs 3, 4), chelicerae with acute cheliceral apophysis at base of fang (Figs 47–49); heavy right-angled embolus (Figs 7, 14, 27); female with small posterolateral corner of preanal scutum (Fig. 19).

##### Compostion.

Twenty-five species were known before the present publication, eight of which are only known from one sex (World Spider Catalog 2018).

##### Distribution.

Borneo (7 species), Caroline Islands (1), China (1), Indonesia, Sumatra (3), Indonesia, Sulawesi (1), Japan (1), Malaysia (2), New Guinea (5), Philippines (2), Singapore (1), Solomon Island (1) and Thailand (1).

#### Ablemma andriana

Taxon classificationAnimaliaAraneaeTetrablemmidae

Fardiansah & Dupérré sp. n.

http://zoobank.org/2ABD581D-721B-4CCC-B15E-50A66D95CA5F

Figs 1–4 , 5–7 , 47

##### Type material.

**Holotype** ♂: Indonesia, Sumatra, Harapan, 02°11'15.2"S, 103°20'33.4"E, secondary lowland rainforest, October 2012, M. Jochum, A. Barnes (LIPI). **Paratypes**: 1♂, same data as holotype (LIPI); Indonesia, Sumatra, Harapan, 02°09'09.9"S, 103°21'43.2"E, secondary lowland rainforest, 3♂, October 2012, M. Jochum, A. Barnes (LIPI) (ZMH–A0001339); 02°09'29.2"S, 103°20'01.5"E, secondary lowland rainforest, 1♂, October 2012, M. Jochum, A. Barnes (ZMH–A0001346); 02°09'09.9"S, 103°21'43.2"E, secondary lowland rainforest, 1♂, October 2013, B. Klarner (ZMH–A0001221); 1♂, October 2013, B. Klarner (ZMH–A0001222); 1♂, October 2013, B. Klarner (ZMH–A0002644); 01°54'39.5"S, 103°16'00.1"E, rubber plantation, 1♂, October 2013, B. Klarner (ZMH–A0001220); 02°09'09.9S 103°21'43.2"E, secondary lowland rainforest, 1♂, October 2017, B. Klarner (ZMH–A0001212).

**Figures 1–4.**
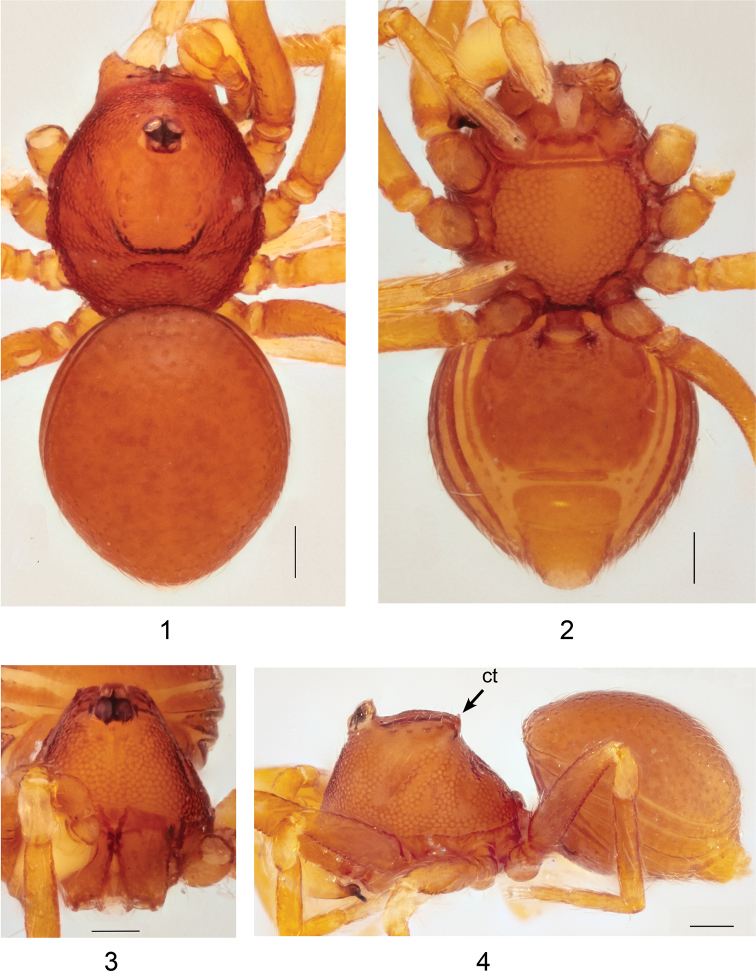
*Ablemmaandriana* sp. n., male. **1** Habitus, dorsal view. **2** Habitus, ventral view. **3** Carapace, frontal view. **4** Habitus, lateral view. Scale bar: 1.0 mm.

##### Etymology.

The specific name is an arbitrary combination of letters.

##### Diagnosis.

**Figures 5–7.**
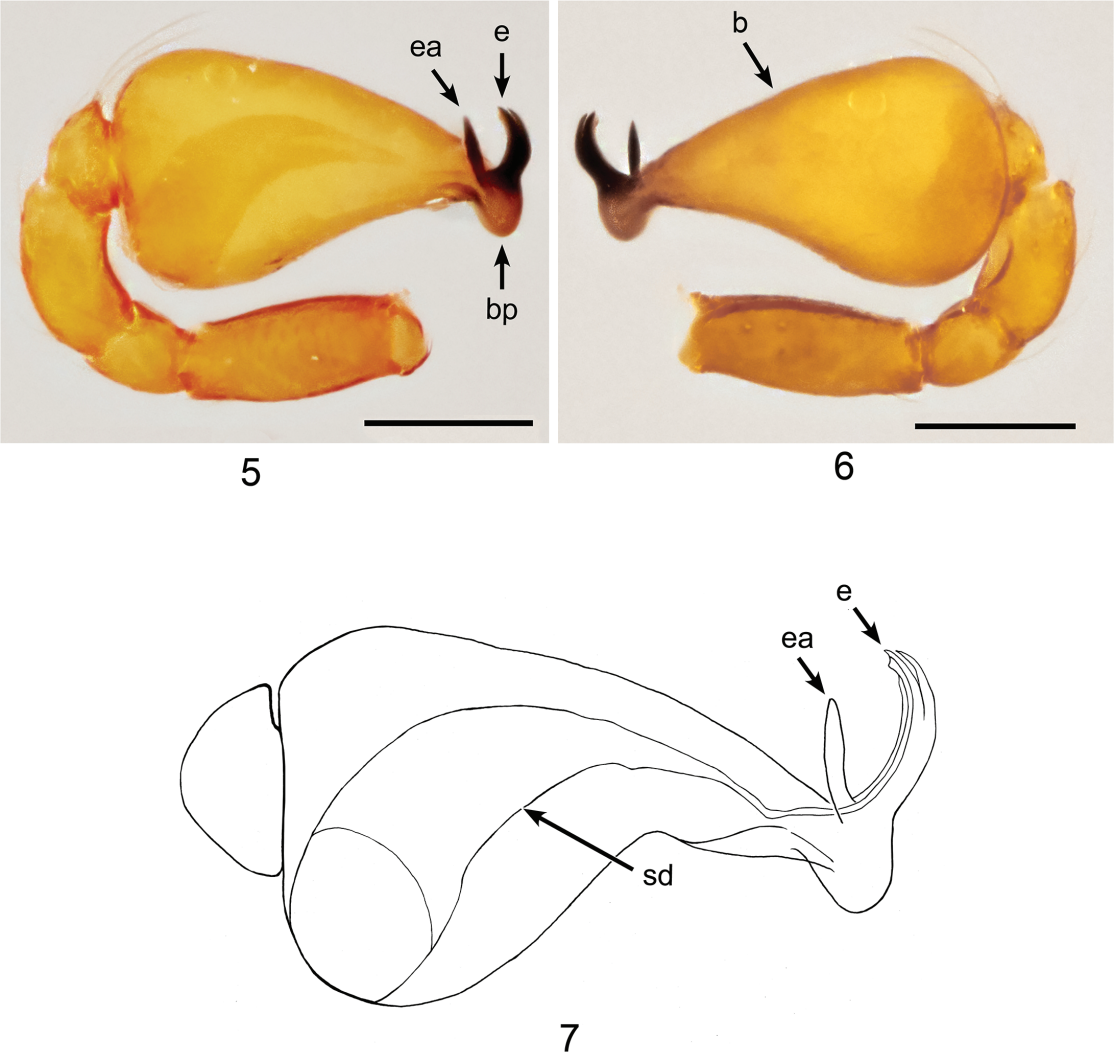
*Ablemmaandriana* sp. n., male. **5** Palp, prolateral view. **6** Palp, retrolateral view. **7** Schematic illustration of palpal bulb retrolateral view. Scale bar: 0.1 mm.

Males of *Ablemmaandriana* sp. n. can be distinguished from most *Ablemma* species with the exception *A.erna* Lehtinen, 1981, *A.merotai* Lehtinen, 1981 and *A.malacca* Lin and Li, 2017, by the form of the embolus (Figs 5–7) and the cheliceral boss (Fig. 47). From *A.erna* and *A.malacca* it can be separated by the bifid apical end of the embolus (Figs 5–7); the embolus of the latter species is not split (see Lehtinen 1981, figs 171-172; Lin and Li et al. 2017, figs 8B, C). From *A.merotai* males are differentiated by their rounded cheliceral boss bearing a small tubercle (Fig. 47), meanwhile the chelicera basal boss is weakly developed in latter species (see Lehtinen 1981, fig. 163).

##### Description.

***Male (holotype)*. Measurements**: Total length: 1.03; carapace length: 0.44; carapace width: 0.40; abdomen length: 0.59; abdomen width: 0.44; clypeus height: 0.19. Length of legs: I 0.97 (0.33, 0.11, 0.24, 0.13, 0.16); II 0.95 (0.31, 0.11, 0.21, 0.16, 0.16); III 0.82 (0.26, 0.09, 0.18, 0.15, 0.15); IV 1.12 (0.36, 0.11, 0.28, 0.19, 0.18).

*Carapace*: Brownish orange; pars cephalica smooth apically, slightly concave; apico-laterally with a fringe of 5 pits, each bearing setae, and two small tubercles at apical end; sides finely reticulated; pars thoracica finely reticulated, sloping gradually (Figs 1, 4); clypeus brownish orange; sloping forward; finely reticulated (Figs 3, 4). *Sternum*: Brownish orange; slightly wider than long; reticulated except along midline (Fig. 2). *Chelicerae*: Brownish orange; cheliceral basal boss curved, bearing one small tubercle; cheliceral apophysis large and triangular; transparent lamina thin (Fig. 47). *Eyes*: 6 in compact group (Fig. 1). *Abdomen*: Dorsal scutum brownish orange, smooth, covered with setae (Fig. 1). Laterally with 4 brownish orange stripe-like scuta, slightly separated (Fig. 4). Ventrally covered by 4 smooth scuta: pulmonary scutum brownish orange, with darker, oval book-lung covers; postgenital scutum small and straight, shorter than preanal scutum; preanal scutum large, rectangular; anal scutum conical, short and curved. *Legs*: Brownish orange; femora I–IV rugose ventrally; tibia I with small ventral tubercle; tarsus I swollen. *Palp*: Segments yellowish brown. Cymbium short, as long as patella; bulb yellowish white, pyriform (Figs 5, 6). Sperm duct thick, narrowing with sharp angle; embolus dark, sharp, slightly bifid apically with spine-like embolic apophysis; basal projection almost as long as embolus (Fig. 7).

***Female*.** Unknown.

##### Natural history.

Most specimens were collected in secondary lowland rainforest; one specimen was collected in a rubber plantation.

##### Distribution.

Known only from the type locality, Harapan, Sumatra.

#### Ablemma contrita

Taxon classificationAnimaliaAraneaeTetrablemmidae

Fardiansah & Dupérré sp. n.

http://zoobank.org/6AE341BC-2AAE-4F6C-A06E-B7BCEBAB9CCF

Figs 8–11 , 12–14 , 15–18 , 19–20 , 48

##### Type material.

**Holotype** ♂: Indonesia, Sumatra, Harapan, 01°49'31.9"S, 103°17'39.2"E, jungle rubber, October 2012, M. Jochum, A. Barnes (LIPI). **Paratypes** 1♂ and 1♀, same data as holotype (ZMH–A0001219).

##### Etymology.

The specific name is a noun in apposition taken from Latin, meaning *broken*, in reference to the tip of the embolus.

##### Diagnosis.

**Figures 8–11.**
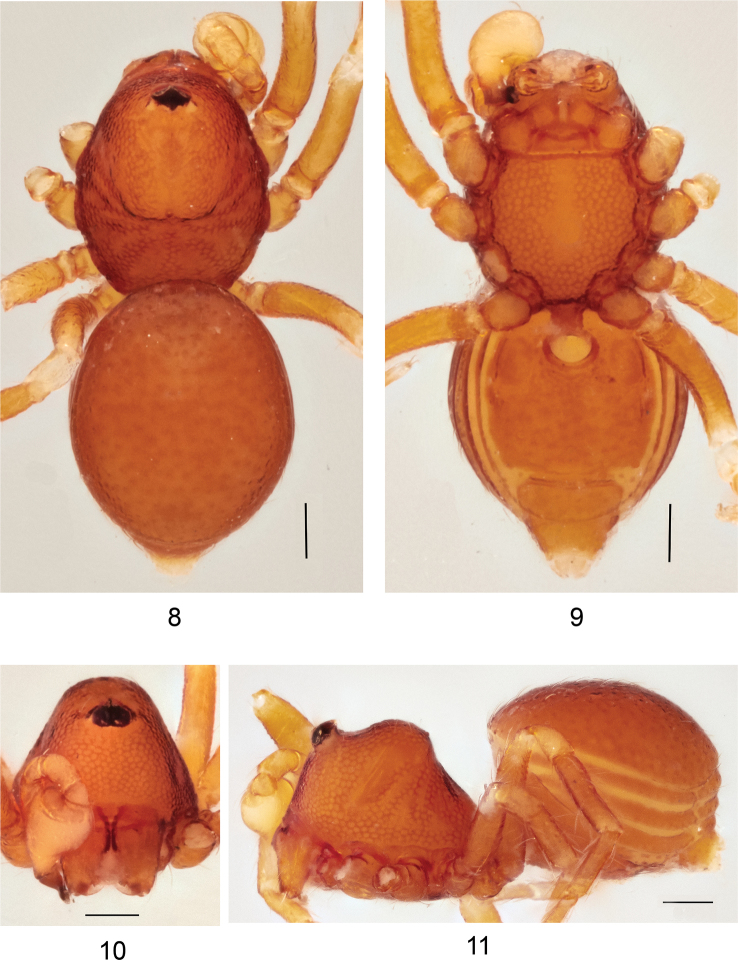
*Ablemmacontrita* sp. n., male. **8** Habitus, dorsal view. **9** Habitus, ventral view. **10** Carapace, frontal view. **11** Habitus, lateral view. Scale bar: 1.0 mm.

**Figures 12–14.**
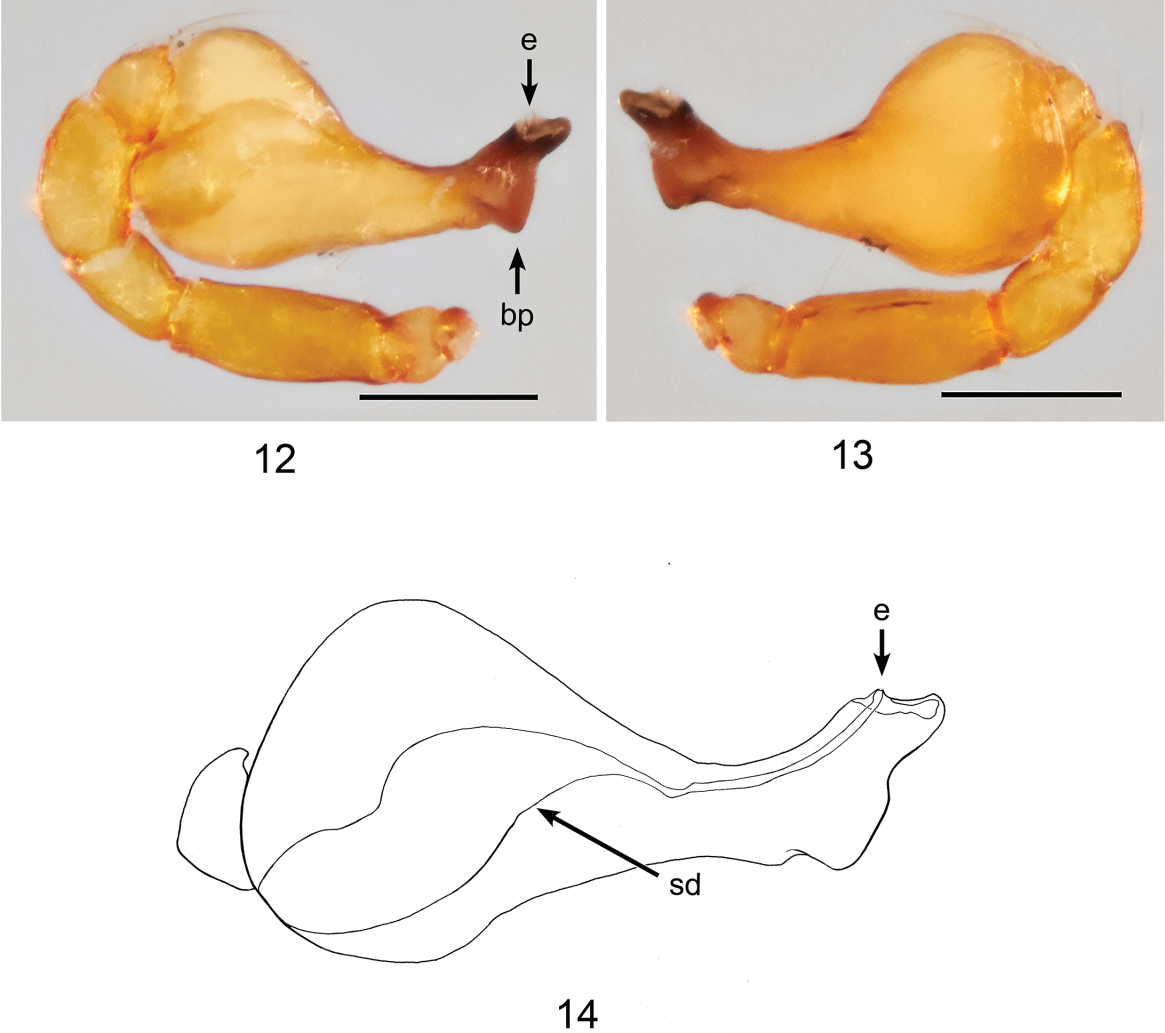
*Ablemmacontrita* sp. n., male. **12** Palp, prolateral view. **13** Palp, retrolateral view. **14** Schematic illustration of palpal bulb, retrolateral view. Scale bar: 0.1 mm.

Males can be distinguished from all congeners with the exception of similar species (*A.unicornis* Burger, 2008, *A.erna*, *A.kaindi* Lehtinen, 1981, and *A.malacca* by the blunt apical end of the male embolus (Figs 12, 13). Furthermore, *A.contrita* sp. n. differ from *A.unicornis* by the lack of an anterior tooth on the carapace behind the eye group (Figs 8, 11) vs. present in *A.unicornis* (see Burger 2008, 254, figs 1, 2); from *A.erna* and *A.kaindi* (see Lehtinen 1981, 128-129, figs 166, 171) by its shorter and flatter embolus, and the more elongated palpal bulb (Figs 12, 13); from *A.malacca* by the concave and swollen pars cephalica (Fig. 11), straight and not swollen in the latter (see Lin et al. 2017, fig. 7). Females are distinguished from similar species as follows: from *A.unicornis* by the absence of the posterior pit on the sternum in females (Fig. 16); from *A.kaindi* by their longer inner genitalic plate; from *A.erna* by their wider, not expanded apically inner gentitalic plate; from *A.malacca* by the shorter and thicker inner gentitalic plate (Fig. 20).

##### Description.

***Male (holotype)*.** Measurements: Total length: 0.91; carapace length: 0.41; carapace width: 0.35; abdomen length: 0.50; abdomen width: 0.40; clypeus height: 0.16. Length of legs: I 0.88 (0.28, 0.11, 0.22, 0.12, 0.15); II 0.75 (0.24, 0.10, 0.16, 0.12, 0.13); III 0.74 (0.22, 0.10, 0.16, 0.13, 0.13); IV 0.92 (0.28, 0.12, 0.22, 0.16, 0.15).

*Carapace*: Brownish orange, pars cephalica finely reticulated, concave and then strongly convex with one small tubercle at apical end; sides finely reticulated; pars thoracica reticulated, sloping gradually (Figs 8, 11); clypeus brownish orange; sloping forward; finely reticulated (Figs 10, 11). *Sternum*: Brownish orange; slightly wider than long; reticulated except along midline (Fig. 9). *Chelicerae*: Brown; cheliceral basal boss straight, thick; cheliceral apophysis small, triangular; lamina thin, transparent (Fig. 48). *Eyes*: 6 in compact group (Fig. 8). *Abdomen*: Dorsal scutum yellowish orange, smooth, covered with setae (Fig. 8). Laterally covered by 4 brownish orange stripe-like scuta, slightly separated (Fig. 11). Ventrally covered by 4 smooth scuta; plumonary scutum brown, with oval book-lung covers; postgenital scutum small and straight, shorter than preanal scutum; preanal scutum large, rectangular; anal scutum triangular conical, short and curved. *Legs*: Yellowish brown, femora I–IV rugose ventrally; tarsi I swollen. *Palp*: Segments yellowish white; bulb pyriform (Figs 12, 13). Sperm duct thick, narrowing gradually; embolus dark brown, apically semi-transparent and truncated; basal projection somewhat triangular (Fig. 14).

***Female (paratype)*.** Measurements: Total length: 0.88; carapace length: 0.42; carapace width: 0.33; abdomen length: 0.46; abdomen width: 0.38; clypeus height: 0.9. Length of legs: I missing; II 0.73 (0.22, 0.11, 0.16, 0.12, 0.12); III 0.66 (0.22, 0.09, 0.15, 0.09, 0.11); IV missing.

**Figures 15–18.**
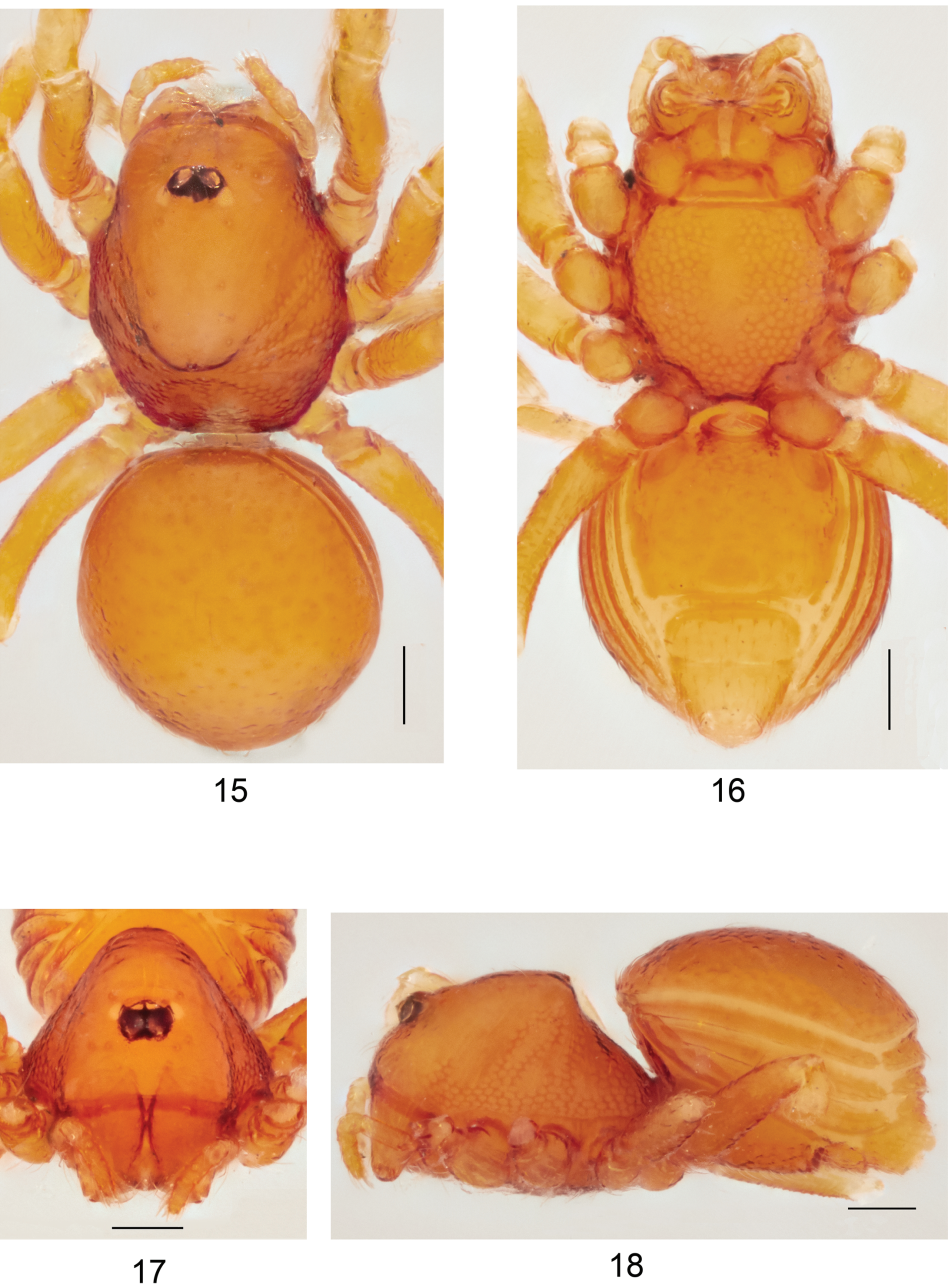
*Ablemmacontrita* sp. n., female. **15** Habitus, dorsal view. **16** Habitus, ventral view. **17** Carapace, frontal view. **18** Habitus, lateral view. Scale bar: 1.0 mm.

**Figures 19, 20.**
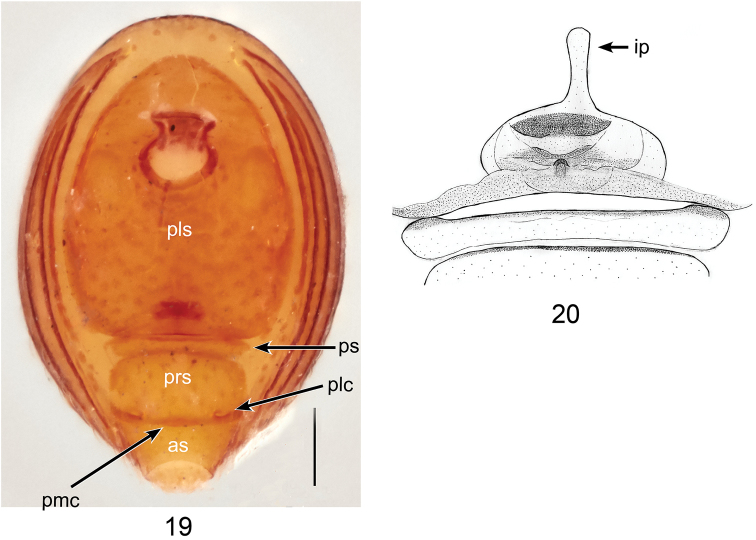
*Ablemmacontrita* sp. n., female. **19** Abdomen, ventral view. **20** Schematic illustration of genitalia, dorsal view. Scale bar: 0.1 mm.

Coloration: Same as in male. *Carapace*: Pars cephalica smooth dorsally, slightly elevated in lateral view, reticulated laterally; pars thoracica reticulated, sloping gradually (Figs 15, 18); clypeus smooth, slightly convex (Figs 17, 18). *Chelicerae*: Brownish orange, not modified. *Eyes*: 6 in compact group, not protruding (Figs 15, 17). *Legs*: Coloration as in male; most legs missing. *Abdomen*: Dorsal and lateral scuta as in male (Figs 15, 18). Ventrally with 4 smooth scuta; pulmonary scutum with oval book-lung covers; postgenital scutum small and straight, shorter than preanal scutum; preanal scutum large, rectangular, with 2 posterolateral corners and small posteromedial corner; anal scutum triangular conical, short and curved (Fig. 19). *Genitalia*: Inner plate short and thick, distal end slightly rounded and wide; lateral horns and ducts not observed; seminal receptacle not observed (Fig. 20).

##### Natural history.

Specimens were only collected in lowland rainforest with rubber trees.

##### Distribution.

Known only from the type locality, Harapan, Sumatra.

#### Ablemma kelinci

Taxon classificationAnimaliaAraneaeTetrablemmidae

Fardiansah & Dupérré sp. n.

http://zoobank.org/E115D44A-5527-4BA2-8B94-A931958F8D64

Figs 21–24 , 25–27 , 49

##### Type material.

**Holotype** ♂: Indonesia, Sumatra, Bukit Duabelas, 01°58'55.1"S, 102°45'02.07"E, secondary lowland rainforest, October 2012, M. Jochum, A. Barnes (LIPI). **Paratype**: 1♂, Indonesia, Sumatra, Bukit Duabelas; 01°59'42.2”S, 102°45'08.01”E, secondary lowland rainforest, October 2012, M. Jochum, A. Barnes (ZMH–A0001340).

##### Etymology.

The specific name is a noun in apposition taken from Indonesian official language «*bahasa*», meaning rabbit, in reference to the eye projection that resembles rabbit ears.

##### Diagnosis.

**Figures 21–24.**
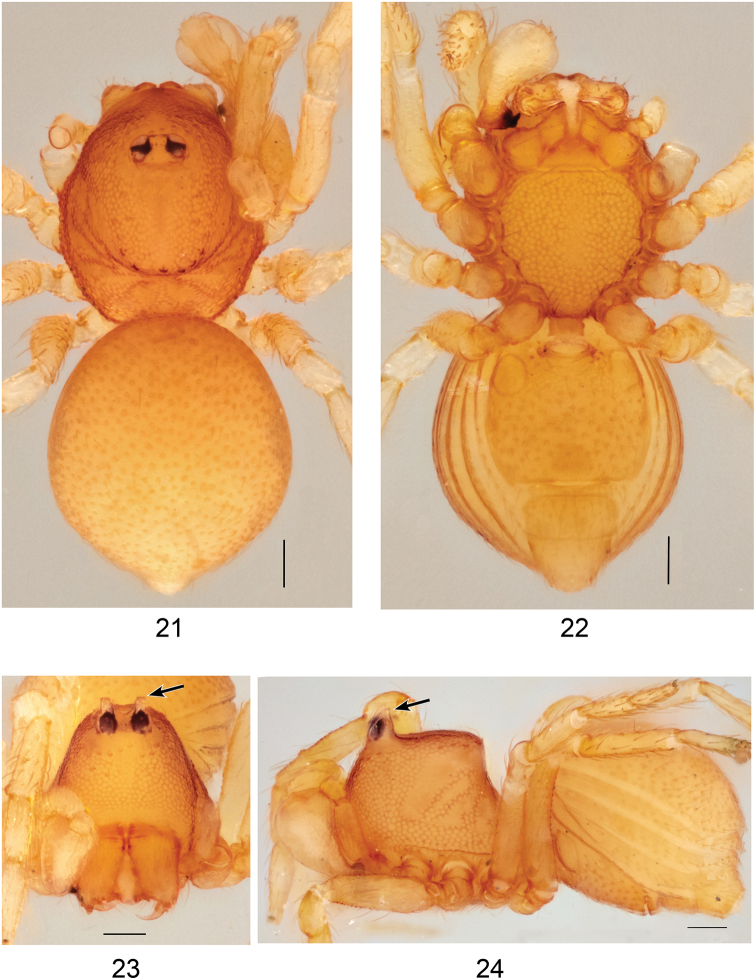
*Ablemmakelinci* sp. n., male. **21** Habitus, dorsal view. **22** Habitus, ventral view. **23** Carapace, frontal view. **24** Habitus, lateral view. Scale bar: 1.0 mm.

Males of *Ablemmakelinci* sp. n. can be distinguished from most species by the presence of only four eyes (Fig. 21), from other four eyed *Ablemma*; *A.sternofoveata* by the PME much closer (see Lethinen 1981, fig. 131); from *A.berryi* Shear, 1978 and *A.shimojamai* by the absence of large apical dorsal carapace tubercle (see Shear 1978, figs 81, 89). Furthermore, males are differentiated from all species by the unique triangular projection above the eyes (Figs 23, 24, arrow).

##### Description.

***Male (holotype)*.** Measurements: Total length: 1.06; carapace length: 0.47; carapace width: 0.42; abdomen length: 0.59; abdomen width:0.51; clypeus height: 0.22. Length of legs: I 1.14 (0.38, 0.12, 0.29, 0.15, 0.20); II 1.02 (0.30, 0.13, 0.25, 0.16, 0.18); III 0.89 (0.27, 0.12, 0.18, 0.16, 0.16); IV 1.21 (0.39, 0.13, 0.30, 0.20, 0.19).

**Figures 25–27.**
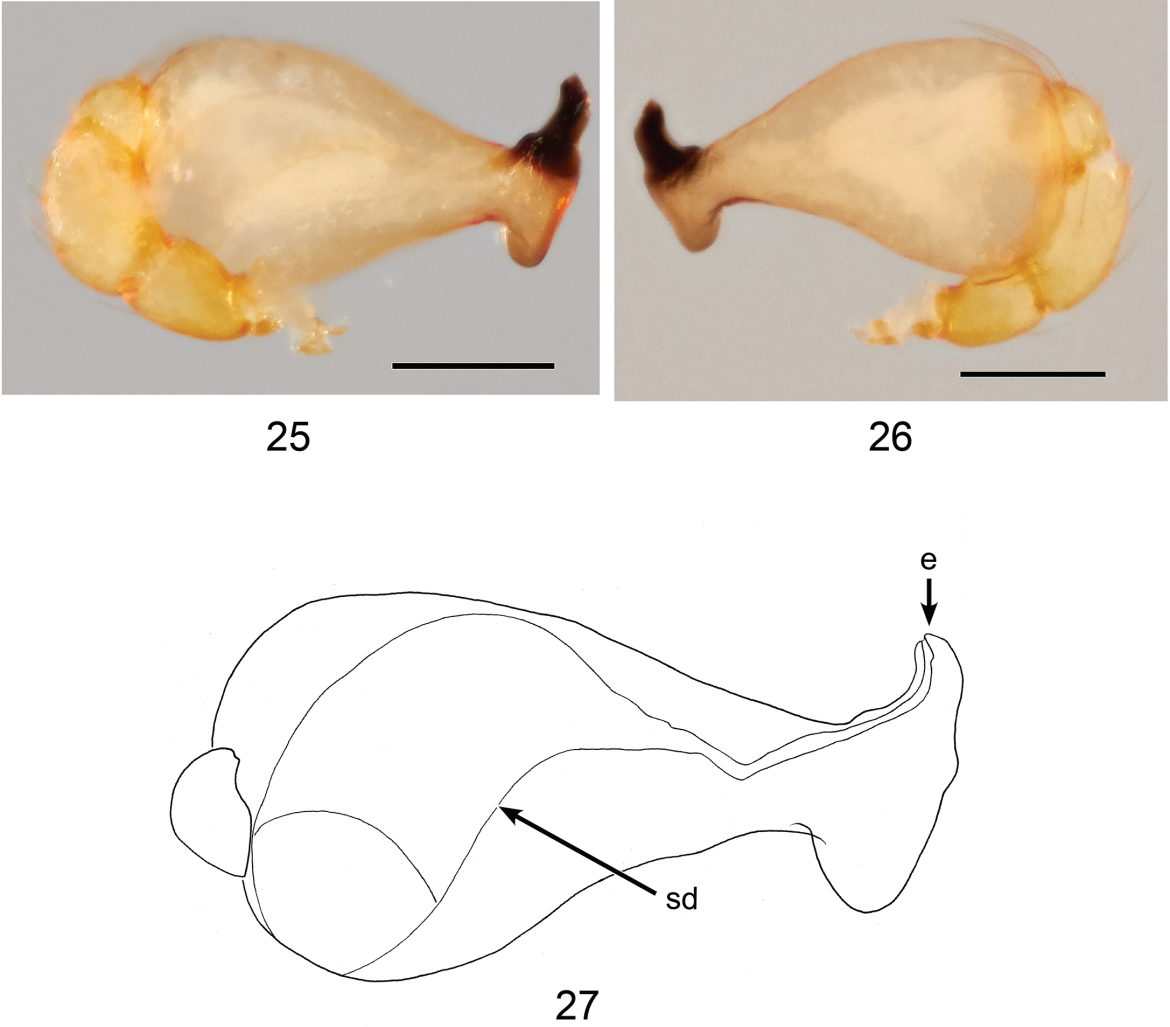
*Ablemmakelinci* sp. n., male. **25** Palp, prolateral view. **26** Palp, retrolateral view. **27** Schematic illustration of palpal bulb retrolateral view. Scale bar: 0.1 mm.

*Carapace*: brownish yellow; pars cepalica reticulated, slightly concave apically; apico-laterally with a fringe of 5 pits, each bearing setae, and 3 small tubercles at apical end; pars thoracica finely reticulated, steeply sloping (Figs 21, 24); clypeus brownish yellow; sloping forward; finely reticulated (Figs 23, 24). *Sternum*: Yellowish brown; slightly longer than wide; reticulated except medially (Fig. 22). *Chelicerae*: Brownish orange; cheliceral basal boss straight, bearing one small tubercle; cheliceral apophysis large and triangular; transparent lamina thick apically (Fig. 49). *Eyes*: 4 eyes on a small mount; AME separated by their diameter, PME separated by 2× their diameter; AME and PME touching; with triangular projection above eyes (Figs 21, 23 arrow). *Abdomen*: Dorsal scutum yellowish brown, smooth (Fig. 21). Laterally with 4 yellowish brown stripe-like scuta, slightly separated (Fig. 24). Ventrally covered by 4 smooth scuta; pulmonary scutum yellow orange, with oval book-lung covers; postgenital plate scutum and straight rectangular; preanal scutum nearly same width as postgenital scutum small and straight, shorter than preanal scutum; preanal scutum large, rectangular; anal scutum conical (Fig. 22). *Legs*: Yellowish brown; femora I–IV rugose ventrally; tibia I with small ventral tubercle; tarsus I swollen. *Palp*: Segments yellowish white; bulb mostly white, pyriform (Figs 25, 26). Sperm duct thick, narrowing with acute angle; embolus dark, blunt; basal projection thick and rounded (Fig. 27).

***Female*.** Unknown.

##### Natural history.

So far, specimens were collected only from secondary lowland rainforest and never from modified forests such as rubber or oil palm plantations.

##### Distribution.

Known only from the type locality, Bukit Duabelas, Sumatra.

#### Ablemma singalang

Taxon classificationAnimaliaAraneaeTetrablemmidae

Lehtinen, 1981

Figs 28–31 , 32–33

##### Type material.

**Holotype** ♂ and **paratype** ♂ from Indonesia, Sumatera Barat, Padangpanjang district, Gunung Singgalang (1750 m), very dark dense jungle with ferns, 25.IX.1978, PTL, in MZT (Turku); not examined.

##### Additional material examined.

Indonesia, Sumatra, Bukit Duabelas 01°59'42.5"S, 102°45'08.1"E, secondary lowland rainforest litter, 1♀, October 2012, M. Jochum, A. Barnes (LIPI); 1♀, October 2013 (LIPI); Harapan, 02°09'09.9"S, 103°21'43.2"E, secondary lowland rainforest litter, 1♂, October 2012, M. Jochum, A. Barnes (ZMH–A0001347); 1♀, October 2012, M. Jochum, A. Barnes (ZMH–A0001353); 1♀, October 2012, M. Jochum, A. Barnes (ZMH–A0001357); 1♂, October 2012, M. Jochum, A. Barnes (ZMH–A0001358); 1♀, October 2012, M. Jochum, A. Barnes (ZMH–A0002639); 1♂, October 2013 (LIPI); 1♂, October 2013, B. Klarner (ZMH–A0002636); 1♀, October 2013, B. Klarner (ZMH–A0002641); 1♂1♀, 08 March 2017, B. Klarner (ZMH–A0001211); 1♂, 10 June 2017, B. Klarner (ZMH–A0001210); 1♂, 10 June 2017, B. Klarner (ZMH–A0001213); 1♂1♀, 08 March 2017, B. Klarner (ZMH–A0001207); 2♂, 08 August 2017, B. Klarner (ZMH–A0001208); 1♂2♀, October 2017, B. Klarner (ZMH–A0001214); 1♀, October 2017, B. Klarner (ZMH–A0001217); 1♂2♀, 26 November 2017, B. Klarner (ZMH–A0001209); 01°54'39.5"S, 103°16'00.1"E, rubber plantation, 1 ♂, October 2012, M. Jochum, A. Barnes (ZMH-A0001356); 1♀, October 2013, B. Klarner (ZMH–A0002637); 1♂, October 2013, B. Klarner (ZMH–A0002643); 1♂, 26 November 2017, B. Klarner (LIPI); 01°55'40.0"S, 103°15'33.8"E, 1♂, rainforest with rubber trees, 1♂2♀, October 2012, M. Jochum, A. Barnes (ZMH–A0001359); 3♂, October 2012, M. Jochum, A. Barnes (ZMH–A0001360); 1♀, October 2012, M. Jochum, A. Barnes (ZMH–A0002635); 1♂, October 2013, B. Klarner (ZMH–A0001216); 3♂, October 2013, B. Klarner (ZMH–A0002638); 3♂, October 2013, B. Klarner (ZMH–A0002640); 1♀, October 2013, B. Klarner (ZMH–A0002642).

##### Diagnosis.

Females of *Ablemmasingalang* can be distinguished from most congeners by the small rounded basal boss on the chelicerae (Fig. 30, arrow), and the elongated narrow inner plate of the internal genitalia (Fig. 33); short and triangular in other species. Furthermore, females are distinguished from *A.malacca* with similar internal genitalia, by the larger carapace tubercles (Fig. 31), smaller in the latter species (see Lin et al. 2017, fig. 7H).

##### Description.

***Male.*** See Lethinen 1981: 48.

***Female.*** Measurements: Total length: 1.14; carapace length: 0.48; carapace width: 0.41; abdomen length: 0.66; abdomen width: 0.60; clypeus height: 0.11. Length of legs: I 0.90 (0.29, 0.12, 0.23, 0.13, 0.13); II 0.78 (0.28, 0.13, 0.22, 0.15, 0.15); III 0.89 (0.26, 0.11, 0.20, 0.16, 0.16); IV 1.18 (0.36, 0.13, 0.30, 0.20, 0.19).

**Figures 28–31.**
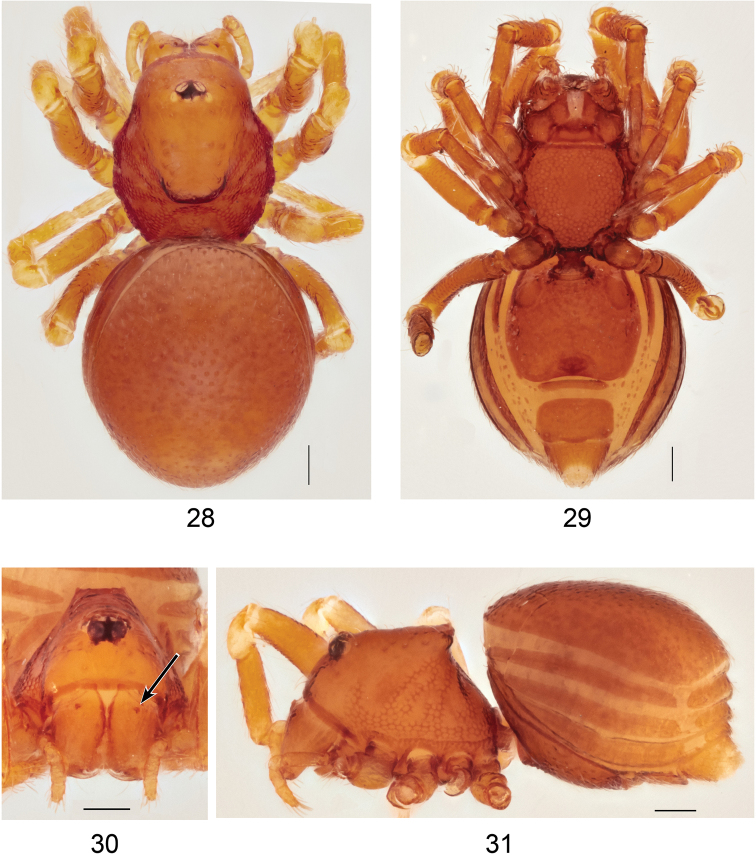
*Ablemmasingalang* Lehtinen, 1981, female. **28** Habitus, dorsal view. **29** Habitus, ventral view. **30** Carapace, frontal view. **31** Habitus, lateral view. Scale bar: 1.0 mm.

**Figures 32, 33.**
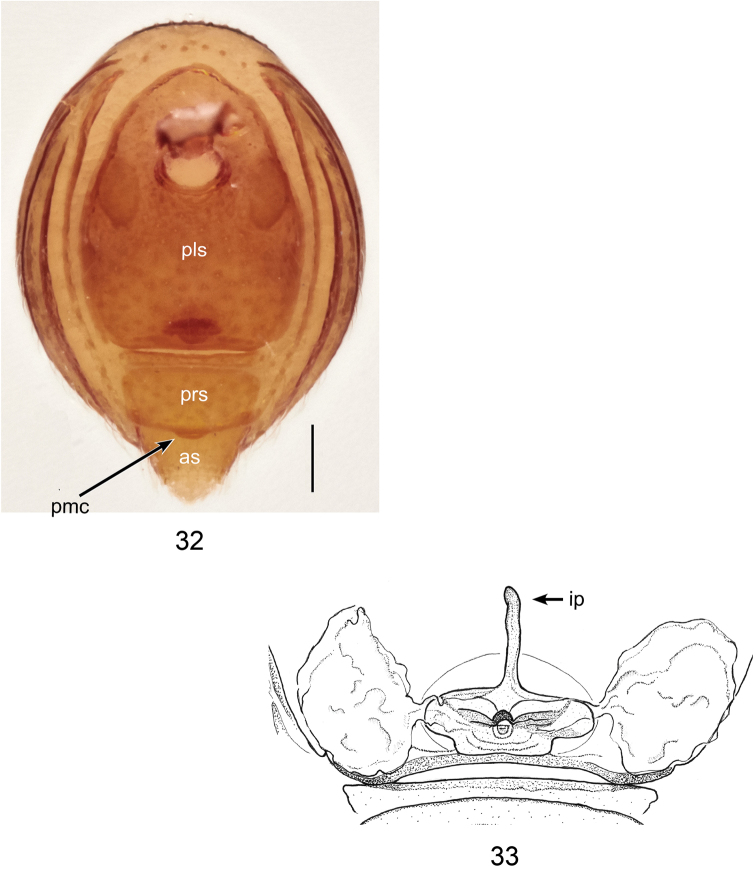
*Ablemmasingalang* Lehtinen, 1981, female. **32** Abdomen, ventral view. **33** Schematic illustration of genitalia, dorsal view. Scale bar: 0.1 mm.

*Carapace*: Brownish orange; pars cephalica smooth dorsally, flat behind the ocular area than sloping upward with a fringe of 5 pits of which each bear a seta, and 3 small tubercles at apical end, reticulated laterally; pars thoracica finely reticulated, sloping straight down and then at an angle (Figs 28, 31); clypeus brownish yellow; sloping forward; short and smooth (Figs 30, 31). *Sternum*: Brownish orange; slightly wider than long, reticulated except medially (Fig. 29). *Chelicerae*: Yellowish orange; basal boss as a small rounded projection (Fig. 30, arrow); lamina well developed, translucent. *Eyes*: 6 eyes in compact group (Fig. 28). *Abdomen*: Dorsal scutum smooth, yellowish brown (Fig. 28). Laterally with 4 yellowish brown stripe-like plate, separated (Fig. 31). Ventrally covered by 4 smooth scuta; plumonary scutum brownish orange, with oval book-lung covers; postgenital scutum thin and straight, shorter than preanal scutum; preanal scutum large, rectangular, with dark and rounded posteromedial corner and 2 small posterolateral corners; anal scutum conical, short and curved (Fig. 32). *Legs*: Brownish orange, femora I–IV rugose ventrally. *Genitalia*: Inner plate long, distal end slightly bent; ducts narrow, transculent, connected to seminal receptacle; sac-like seminal receptacle with distinctly folded cuticle (Fig. 33).

##### Natural history.

Male and females specimens were collected together in secondary lowland rainforest, rainforest with rubber trees, and rubber plantation.

##### Distribution.

Indonesia, Sumatra Barat, Jambi provinces.

#### Genus Brignoliella

Taxon classificationAnimaliaAraneaeTetrablemmidae

Shear, 1978

##### Type species.

*Brignoliellaacuminata* (Simon, 1889)

##### Diagnosis.

**Figures 34–37.**
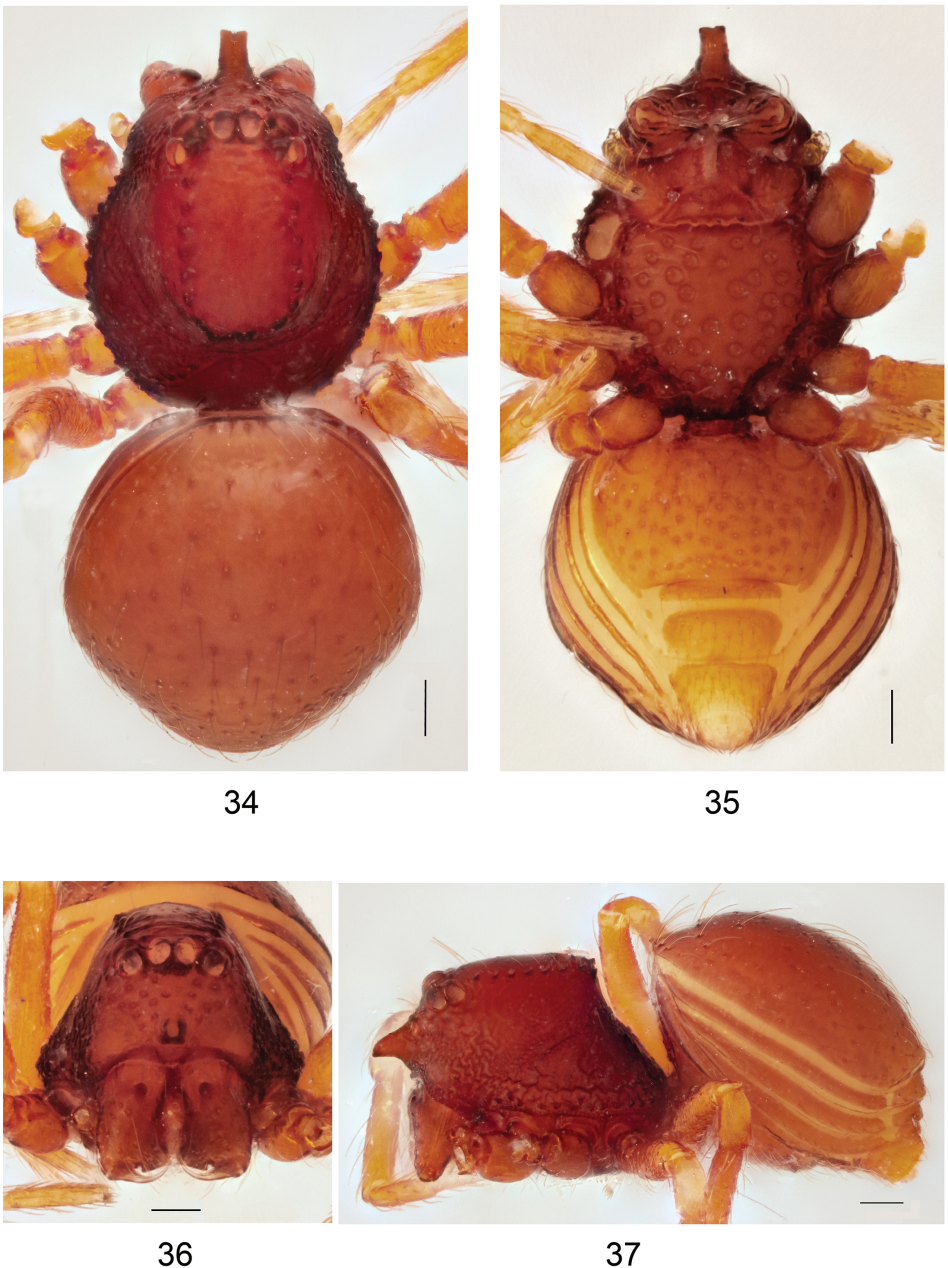
*Brignoliellapatmae* sp. n., male. **34** Habitus, dorsal view. **35** Habitus, ventral view. **36** Carapace, frontal view. **37** Habitus, lateral view. Scale bar: 1.0 mm.

**Figures 38–40.**
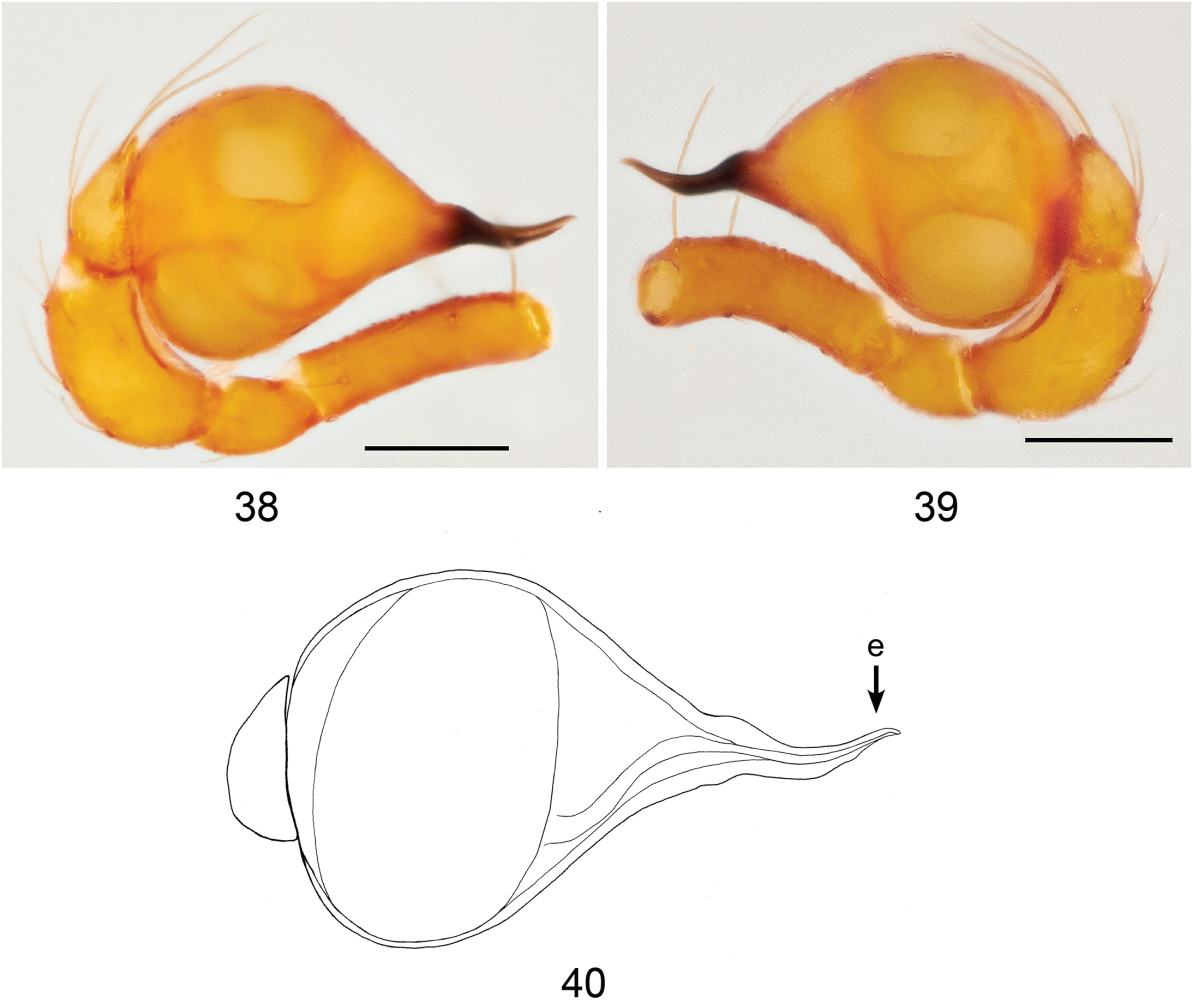
*Brignoliellapatmae* sp. n., male. **38** Palp, prolateral view. **39** Palp, retrolateral view. **40** Schematic illustration of palpal bulb, retrolateral view. Scale bar: 0.1 mm.

The genus *Brignoliella* most resembles the genera *Pahanga* Shear, 1979 and *Borneomma* Deeleman-Reinhold, 1980 but can be distinguished by the combination of the following characters: sternum with regular round punctuations (Fig. 35); male carapace with clypeal horn (Figs 34, 37); embolus sinuous, without terminal acute angle (Figs 38, 39); female with paired anterolateral groove on preanal scutum (Fig. 45).

##### Composition.

Twenty-three species were known before the current publication of which eight are known from one sex only (World Spider Catalog, 2018).

##### Distribution.

Borneo (3 species), Caroline Islands (2), China (2), Fiji (1), India (1), Indonesia Sulawesi (3), Malaysia (2), Nepal (1), New Caledonia (1), New Guinea (1), Papua New Guinea (1), Philippines (2), Singapore (2) and Sri Lanka (2).

#### Brignoliella patmae

Taxon classificationAnimaliaAraneaeTetrablemmidae

Fardiansah & Dupérré sp. n.

http://zoobank.org/55837F24-171D-49F4-A8AC-D2251436D434

Figs 34–37 , 38–40 , 41–44 , 45–46 , 50

##### Type material.

**Holotype** ♂: Indonesia, Sumatra, Harapan, 02°09'29.2"S, 103°20'01.5"E, secondary lowland rainforest, October 2012 (LIPI). **Paratypes**: Indonesia, Sumatra, Bukit Duabelas, 01°56'33.9"S, 102°34'52.7"E, 1♂1♀, secondary lowland rainforest, October 2012 (ZMH-A0001331); 2♀, October 2012 (ZMH-A0001344); 1♂, October 2012 (ZMH-A0001346); 01°59'42.5"S, 102°45'08.1"E, 1♀, secondary lowland rainforest, October 2013 (LIPI); 1♀, October 2013 (ZMH-A000136); 1♀, October 2013 (ZMH-A0001341); Harapan, 02°09'09.9"S, 103°21'43.2"E, secondary lowland rainforest, October 2012 (ZMH-A0001333); 01°49'31.9"S, 103°17'39.2"E, 1♀, rainforest with rubber trees October 2012 (ZMH-A0001351).

##### Etymology.

This species is named after the mother of the first author, Patmawati, nickname “Patma” for her endless support and love.

##### Diagnosis.

This new species can be distinguished from all congeners by the long and narrow embolus, and the shorter length of the clypeal horn (Figs 34, 37). From similar species, *B.besutensis* (see Lin et al. 2017: 33, figs 11A–B) by the longer and thinner embolus, and from *B.michaeli* (see Lehtinen 1981: 119, figs 103–106) by the shorter length of the clypeal horn in males (Figs 34, 37). Females are distinguished from *B.besutensis* and *B.michaeli* (see Lin et al. 2017: fig. 12A–D, fig. 15A–D) by the protruded anterolateral groove of the preanal scutum beyond the anteromargin, and the position of preanal scutum which is separated from the postgenital scutum (Fig. 45).

##### Description.

***Male (holotype).*** Measurements: Measurements: Total length: 1.33; carapace length: 0.58; carapace width: 0.53; abdomen length: 0.75; abdomen width: 0.64; clypeus height: 0.20. Length of legs: I 1.37 (0.43, 0.16, 0.33, 0.22, 0.24); II 1.32 (0.41, 0.16, 0.32, 0.24, 0.24); III 1.21 (0.37, 0.14, 0.26, 0.23, 0.21); IV 1.44 (0.44, 0.14, 0.35, 0.28, 0.23).

*Carapace*: Dark brown, rugose; pars cepalica smooth apically, apico-laterally with a fringe of 7 pits, each bearing setae, and 3 small tubercles at apical end; pars thoracica rugose, steeply sloping (Figs 34, 37); clypeus brown, punctated; clypeal horn fairly long, distally bifid (Figs 34, 37). *Sternum*: Brown; as long as wide; deeply punctated (Fig. 35). *Chelicerae*: Brown, punctated; cheliceral boss a pointed projection; cheliceral apophysis absent; cheliceral lamina well developed, translucent (Figs 36, 50). *Eyes*: 6 eyes in three diads (Fig. 34). *Abdomen*: Dorsal scutum brownish orange, smooth (Fig. 34). Lateral scutum brownish orange, with four pairs of stripe-like plates (Fig. 37). Ventral scutum brownish orange, book-lung cover round; anterior ventrolateral scutum pointed; postgenital scutum rectangular and flat, slightly bend with pulmonary scutum but separated with preanal scutum; preanal scutum slightly square; conical anal scutum triangular (Fig. 35). *Legs*: Brownish orange; femora rugose ventrally; femur I and II slightly swollen; femur IV slightly bent. *Palp*: Bulb yellowish brown, pear-shaped (Figs 38, 39). Embolus dark brownish, sharp and curved (Figs 38–40).

**Figures 41–44.**
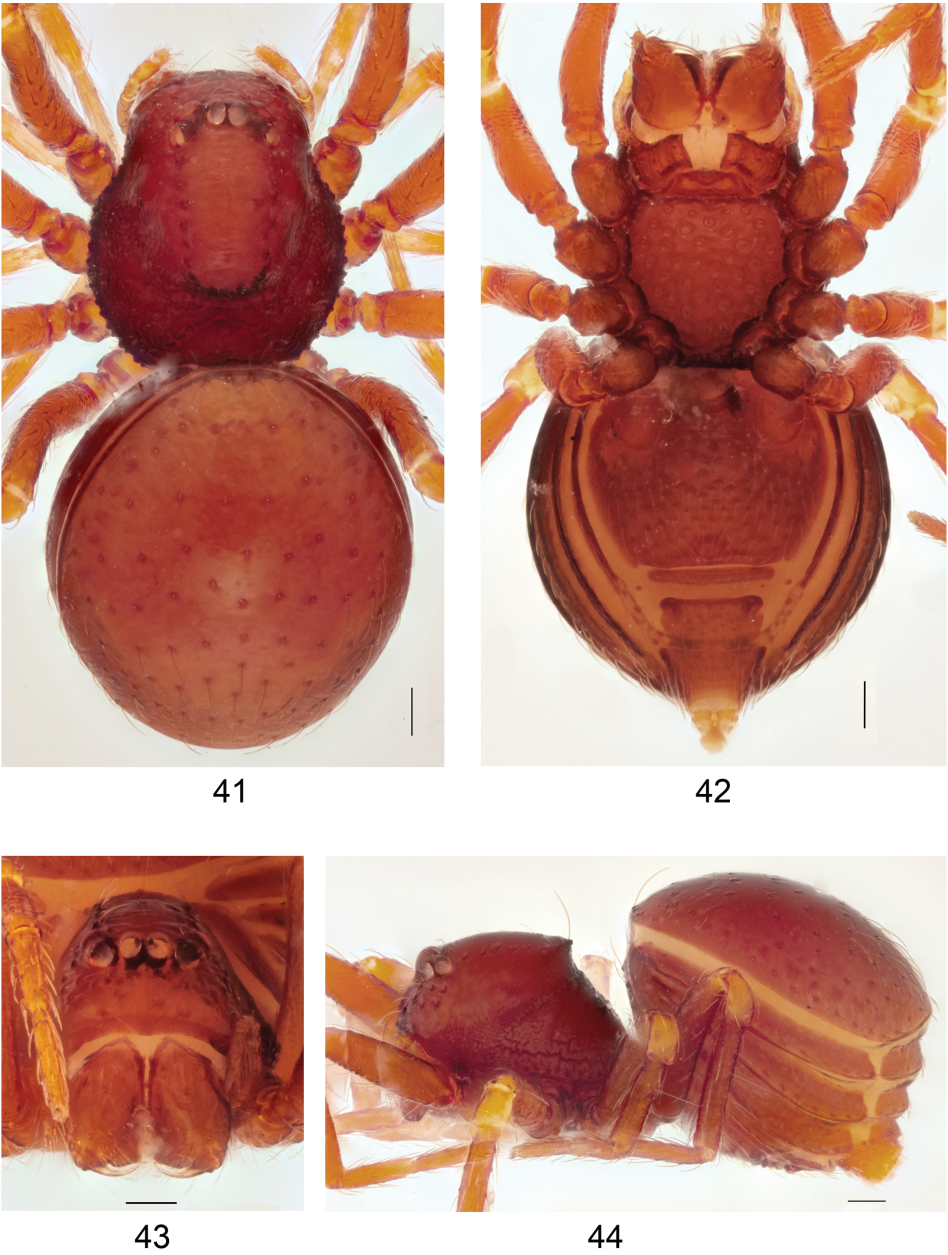
*Brignoliellapatmae* sp. n., female. **41** Habitus, dorsal view **42** Habitus, ventral view. **43** Carapace, frontal view. **44** Habitus, lateral view. Scale bar: 1.0 mm.

***Female (paratype)*.** Measurements: Total length: 1.14; carapace length: 0.56; carapace width: 0.52; abdomen length: 0.87; abdomen width: 0.70; clypeus height: 0.16. Length of legs: I 1.43 (0.49, 0.15, 0.32, 0.22, 0.25); II 1.37 (0.41, 0.16, 0.32, 0.24, 0.24); III 1.17 (0.32, 0.14, 0.26, 0.24, 0.21); 1.56 (0.46, 0.16, 0.39, 0.29, 0.26).

**Figures 45, 46.**
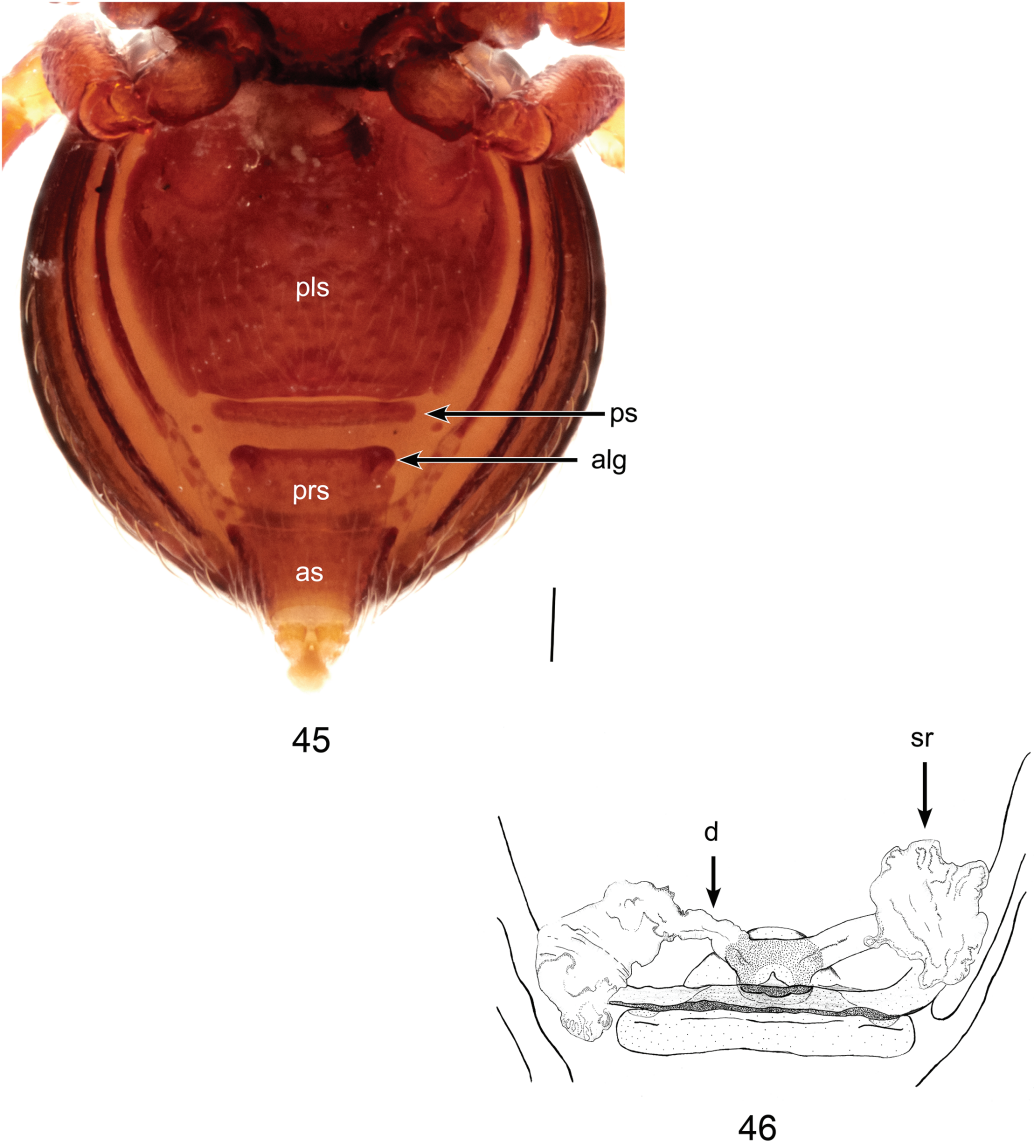
*Brignoliellapatmae* sp. n., female. **45** Abdomen, ventral view. **46** Schematic illustration of genitalia, dorsal view. Scale bar: 0.1 mm.

Coloration: As in male. *Carapace*: Partly rugose, pars cepalica slightly rounded in lateral view; pars thoracica rugose, sloping gradually (Figs 41, 44); clypeus smooth, without clypeal horn, slightly rounded in lateral view (Fig. 44). *Chelicerae*: Cheliceral boss as small rounded projection. *Eyes*: 6 in 3 diads (Figs 41, 43). *Abdomen*: Dorsal scutum brownish orange, smooth (Fig. 41). Laterally with four pairs of stripe-like plates, brownish orange (Fig. 44). Ventrally covered by 4 scuta; pulmonary scutum brownish orange, smooth, book-lungs oval; postgenital scutum slightly straight, longer than preanal scutum; preanal scutum separated from postgenital scutum and anal scutum, anterolateral groove of preanal scutum protruding, rounded, slightly curved and extending beyond the anteromargin; anal scutum triangular conical (Fig. 45). *Legs*: As in male. *Genitalia*: Ducts wide, transculent, connected to seminal receptacle; sac-like seminal receptacle with distinctly folded cuticle (Fig. 46).

##### Natural history.

Secondary lowland rainforest and lowland rainforest with rubber trees.

##### Distribution.

Indonesia, Sumatra.

**Figures 47–50.**
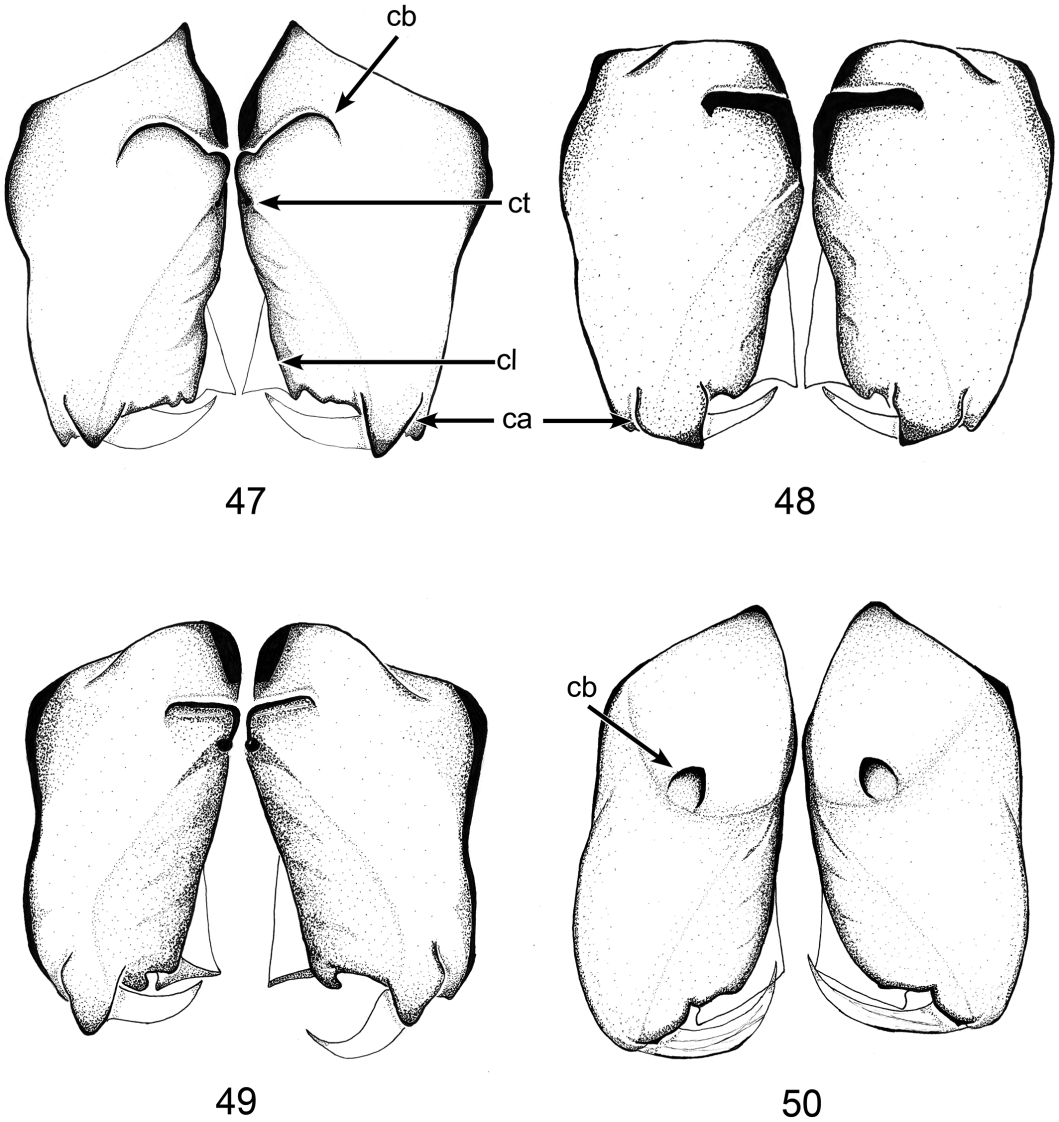
Male chelicerae, anterior views. **47***Ablemmaandriana* sp. n., *A.contrita* sp. n., *A.kelinci* sp. n., and *Brignoliellapatmae* sp. n. **48***A.contrita* sp. n. **49***A.kelinci* sp. n. **50***Brignoliellapatmae* sp. n.

## Supplementary Material

XML Treatment for Ablemma

XML Treatment for Ablemma andriana

XML Treatment for Ablemma contrita

XML Treatment for Ablemma kelinci

XML Treatment for Ablemma singalang

XML Treatment for Brignoliella

XML Treatment for Brignoliella patmae
